# Comparison of whole genomes of tick-borne encephalitis virus from mountainous alpine regions and regions with a lower altitude

**DOI:** 10.1007/s11262-020-01821-w

**Published:** 2021-01-24

**Authors:** G. Lemhöfer, L. Chitimia-Dobler, G. Dobler, M. Bestehorn-Willmann

**Affiliations:** 1 German Center of Infection Research (DZIF) Partner, Bundeswehr Institute of Microbiology, Munich, Germany; 2 Parasitology Unit, Institute of Zoology, University of Hohenheim, Stuttgart, Germany; 3 Department of Veterinary Virology and Microbiology, Ludwig-Maximilians-University (LMU), Munich, Germany

**Keywords:** Tick-borne encephalitis virus, Mountains, Genetic analysis, Tick-borne encephalitis, Tick-borne encephalitis virus strains

## Abstract

Tick-borne encephalitis (TBE) has been a notifiable disease in Germany since 2001. Its causative agent, the TBE virus (TBEV), is the most important arbovirus in Europe and Northern Asia. The illness, caused by the European Subtype usually displays flu-like symptoms, but can result in sequelae and, in 2 % of all cases, in death. Over the last few decades, the virus has spread into new habitats, such as higher altitudes in the Alpine region. For this study, it was hypothesized that the environmental challenges that the virus might be exposed to at such altitudes could lead to the selection of viral strains with a higher resilience to such environmental factors. To determine whether strains identified at higher altitudes possessed different genetic traits compared to viruses from lower altitudes, an analysis of viral genomes from higher Alpine altitudes (> 500 m above sea level) (n = 5) and lower altitudes (< 500 m above sea level) (n = 4) was performed. No common phylogenetic ancestry or shared amino acid substitutions could be identified that differentiated the alpine from the lowland viral strains. These findings support the idea of many individual introductions of TBEV into the alpine region and the establishment of foci due to non-viral specific factors such as favorable conditions for vector species and host animals due to climate change.

## Introduction

TBEV is the agent of tick-borne encephalitis (TBE), a severe infection of the central nervous system that may result in sequelae and can possibly end in disability or death. Currently, the virus is divided into three subtypes, the European, the Siberian and the Far Eastern subtype [1–5]. TBEV is mostly transmitted via tick bites. However, especially in Eastern Europe, outbreaks caused by alimentary transmission through unpasteurized milk and soft cheese have been reported [6–12].

In recent years, the European subtype of TBEV has come more into focus since its distribution pattern changed significantly. The virus appeared in mountainous regions of the Alps previously considered free of natural foci of TBEV [13, 14]. It is unclear, whether certain/specific genetic traits of the virus are responsible for this sudden claim of new endemic areas or if the climatic conditions changed and became more suitable for the natural transmission cycle. It is possible that the mountainous strains were naturally selected due to some small nucleotide polymorphisms (SNPs) in their genome that made them more resistant to the alpine environment. To shed light on the question, 5 different virus strains, isolated from these new endemic areas, were thoroughly analysed regarding their genomic sequences and compared to strains isolated from long established foci in lower altitudes.

## Results and discussion

**Fig. 1:**
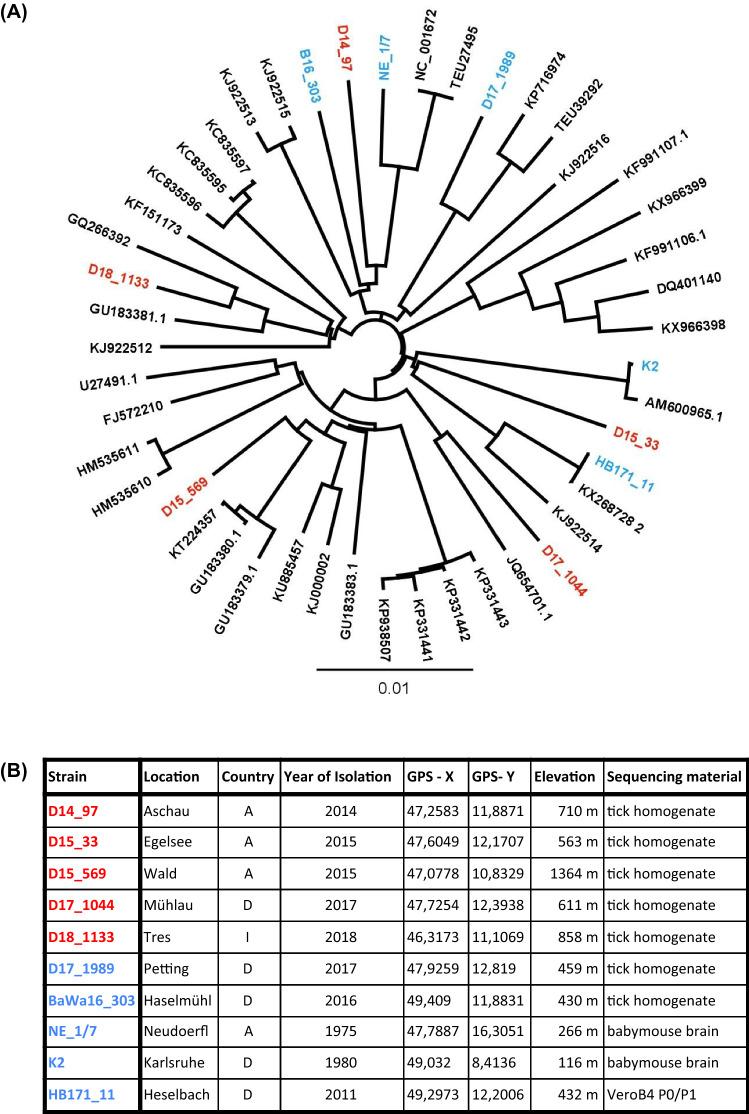
**a** Phylogenetic tree of whole genomes of several TBEV-Eu strains. The whole genomes were amplified in three DNA amplicons covering the whole genome [15]. For sequencing the Illumina MiSeq platform and the MiSeq reagent kit V3 (Illumina, Inc., San Diego, USA) was used, following the manufacturer’s instructions. Assembly was performed using the software Spades v.3.12. For the phylogenetic comparison, available TBEV-Eu whole genome sequences published in the NCBI GenBank database were chosen. The tree was generated using the maximum likelihood approach and 1000 bootstraps were implemented for statistical support [16–19]. The strains used in this study are highlighted in red (alpine regions) and blue (lower altitudes). **b** Table containing the meta data for the TBEV strains used in the analysis

For all TBEV-EU strains included in the study the whole genome sequences were generated. The phylogenetic tree based on the nucleotide sequences showed no evidence of a common origin of the mountainous strains (Fig. 1). The virus strains D15_33, K2 and HB171_11 had the closest phylogenetic relation to each other, despite coming from different elevation levels and collecting sites being 200 km apart.

In Table 1 the different strains have been compared in regard to nucleotide changes/amino acid differences. This analysis reveals that the strain K2 differs the most from strain NE_1/7 (Neudoerfl) with 219 nucleotide changes (shown in *Italics*), whereas the strains D15_569 and D17_1044 show the fewest differences (shown in **Bold**). Regarding amino acid substitutions the biggest difference of 40 amino acid changes is between strains K2 and D17_1989 (highlighted in *Italics*) and the least amino acid difference can be observed between the strains HB171_11 and BaWa16_303 with only 19 amino acid changes (indicated in **Bold**).

**Table 1:** Comparison of nucleotide changes to resulting amino acid changes of all nine virus strains

	D14_97	D15_33	D15_569	D17_1044	D18_1133	D17_1989	BaWa16_303	NE_1/7	K2	HB171_11
D14_97		97,335 (273)	97,648 (241)	97,326 (274)	97,55 (251)	97,374 (269)	97,696 (236)	97,804 (225)	97,306 (276)	97,365 (270)
D15_33	99,268 (25)		97,726 (233)	97,413 (265)	97,599 (246)	97,443 (262)	97,384 (268)	97,394 (267)	97,433 (263)	97,628 (243)
D15_569	99,18 (28)	99,268 (25)		**97,921 (213)**	97,833 (222)	97,657 (240)	97,706 (235)	97,667 (239)	97,677 (238)	97,862 (219)
D17_1044	99,18 (28)	99,209 (27)	99,121 (30)		97,521 (254)	97,433 (263)	97,365 (270)	97,365 (270)	97,345 (272)	97,531 (253)
D18_1133	99,356 (22)	99,356 (22)	99,297 (24)	99,238 (26)		97,531 (253)	97,56 (250)	97,501 (256)	97,54 (252)	97,589 (247)
D17_1989	99,092 (31)	99,151 (29)	99,063 (32)	99,151 (29)	99,092 (31)		97,579 (248)	97,462 (260)	97,267 (280)	97,482 (258)
BaWa16_303	99,385 (21)	99,356 (22)	99,268 (25)	99,268 (25)	99,385 (21)	99,151 (29)		97,823 (223)	97,355 (271)	97,511 (255)
NE_1/7	99,238 (26)	99,209 (27)	99,121 (30)	99,238 (26)	99,238 (26)	99,033 (33)	99,385 (21)		*97,257 (281)*	97,384 (268)
K2	99,092 (31)	99,121 (30)	99,033 (33)	99,033 (33)	99,121 (30)	*98,828 (40)*	99,238 (26)	99,033 (33)		97,609 (245)
HB171_11	99,297 (24)	99,326 (23)	99,18 (28)	99,18 (28)	99,297 (24)	99,063 (32)	**99,443 (19)**	99,238 (26)	99,151 (29)	

The upper right part of the table shows the nucleotide identities given in percentage and the absolute number of snps in brackets. The analysis was performed by the software Geneious 9.1. The lower left part shows the identical projections on amino acid level. For analysis the whole open reading frame was used. The biggest difference is shown in *Italics*, while the strain combination with the least difference is shown in **Bold**

All ten amino acid sequences were aligned and analysed for substitutions. No common amino acid substitution differentiating the alpine and the lowland strains was found. All virus proteins were analysed separately. The capsid protein showed up to two individual changes in the amino acid sequence. The prM/M protein showed up to two individual changes in the sequence with only strain HB171_11 having a non-synonymous change of T141I. The strain D14_97 showed four amino acid changes in the E-gene sequence, two of which were heterologous and may affect the superficial charge of the virus membrane. Other non-synonymous changes were found in strain D15_569 with L459S, strain D17_1989 that has a Y130H and K2 with A83T. Y130H has already been correlated with increased neuroinvasiveness in immunodeficient mice [20].

The NS1 protein showed up to two individual amino acid changes. Strain D17_1044 showed a non-synonymous substitution of the non-polar I127T, K2 one of P103S and HB171_11 A41T. Compared to its length the most variable protein was NS2A. The three strains D15_33, K2 and HB171_11 showed the same synonymous substitution of V41I and the strains D17_1044 and D17_1989 shared a substitution of I53M. The latter is remarkable, since both belong to two different genetic clusters and share another substitution in their NS3 protein.

Within the NS2B amino acid alignment two non-synonymous changes could be identified for the strains D15_569 and HB171_11. For NS3 up to three individual substitutions were found and three of the strains (D15_33, D18_1133 and K2) showed non-synonymous changes. The NS4A showed one substitution in three viruses, with only the substitution of Gly100 for serine in strain D17_1044 being non-synonymous. Three strains (D17_1989, K2 and HB171_11) had up to two individual substitutions in the NS4B protein.

The NS5 showed up to fourteen amino acid substitutions ranging from one to seven individual changes. The virus with most individual changes was strain D17_1044, followed by strain D17_1989.

The virus strain with the fewest individual amino acid changes was strain D18_1133 with 23% changes in their amino acid sequence. It was followed by strain D14_97 with 33.3% individual changes. The virus strain with most individual changes was HB171_11 with 48%.

Therefore, a phylogenetic analysis often results in poor statistical support and has to be interpreted very cautiously. The comparison of our TBEV strains confirms this observation, since the genetic difference of the ten TBEV strains in our study was 0.028 substitutions per site in total. Over the last two decades a clear shift in the distribution of TBE virus from lower to higher altitudes has been observed. So far, the possible effects on the genomic characteristics of TBEV are still unclear. One working hypothesis was that the virus replication had to adapt to harsh environmental conditions. Therefore, these strains might exhibit certain specific changes in their genomes as a response to the conditions they were exposed in regions with higher altitudes. Another explanation is that every strain from a mountainous area is derived from a common ancestor adapted to the conditions found in mountains. After further distribution, they could form new natural foci. Furthermore, a change in climatic and therefore eco-epidemiological conditions in the higher altitudes might lead to better conditions for TBEV replication in ticks.

We were not able to find a common specific genetic trait shared by all strains from areas of an altitude above 500 m above sea level. According to the phylogenetic analysis, each virus strain was probably introduced individually into its location and found favourable environmental conditions (possibly due to climate changes). We observed some of the amino acid changes described in the paper by Formanová et al. [21] in all eight virus strains (namely Ile167V, E127D, V201I and G206R). In addition, T33S was found in HB171_11 and D17_1989 and I53M in D17_1044 and D17_1989. Furthermore, we could not determine a close phylogenetic relationship between the mountainous strains. In fact, strain D15_33 was genetically most closely related to the lower altitudinal region strains HB171_11 and K2 in our analysis. This is another indicator for the newly emerged strains to be distributed into new areas by chance.

NS2A is said to be involved in the shift between RNA packaging and RNA replication [22, 23], its high variability could be the reason for the different replication rates and infectivity of different TBEV strains. The same could apply to the high variability in NS2B, a protein suspected to be involved in modulating the membrane permeability during infection [24]. To confirm or disprove this hypothesis further experiments need to be undertaken.

Since we could not find any hints pointing towards specific genetic characteristics of TBEV strains from higher altitudes, we assume that other reasons for the new distributional pattern are responsible for the emerging alpine distribution of TBEV.

As shown by Rubel et al. in [25] the Alps, have undergone a severe climatic shift since 1876. It can be assumed that this shift contributes to transformation of the Alpine environment and therefore, the distribution of vector species, and host animals might change. This has yet to be proven by further research.
